# Efficacy and safety of metformin in combination with chemotherapy in cancer patients without diabetes: systematic review and meta-analysis

**DOI:** 10.3389/fonc.2023.1176885

**Published:** 2023-07-20

**Authors:** Kang Yang, Hao-hao Lu, Wei Zhao, Qingchun Zhao

**Affiliations:** ^1^ Department of Pharmacy, General Hospital of Northern Theater Command of PLA, Shenyang, China; ^2^ China Medical University, Shenyang, China

**Keywords:** metformin, chemotherapy, PFS, OS, meta-analysis

## Abstract

**Background:**

The results of a meta-analysis of retrospective studies suggest that the use of metformin in cancer patients may prolong progression-free disease survival and overall survival. However, the studies included in the meta-analysis did not strictly distinguish between patients with or without type 2 diabetes mellitus. Therefore, further studies are needed to assess whether the use of adjuvant chemotherapy with metformin in cancer patients without diabetes improves prognosis.

**Method:**

Systematic searches of Embase, Pubmed, and The Cochrane library were performed for the subject terms metformin and neoplasm and for free words. Data related to PFS, OS were extracted according to inclusion exclusion criteria. The data were combined and meta-analysis was performed using Review Manager 5.4 to confirm the efficacy and safety of metformin administration.

**Results:**

There were 3228 publications retrieved from the database and a total of 13 publications with 955 patients were included in the meta-analysis after screening. All included studies were randomised controlled trials. Metformin combined with adjuvant chemotherapy did not improve progression-free survival (HR=1,95CI 0.79-1.25), overall survival (HR=0.91,95% CI 0.69-1.20) and did not improve objective disease response rates in patients. There was no significant difference in grade 3-4 adverse reactions compared to placebo.

**Conclusion:**

In this meta-analysis of randomised controlled trial studies, we found that chemotherapy in combination with metformin in cancer patients without diabetes did not prolong progression-free survival and overall survival and improved disease control in patients, although there was no significant difference in terms of safety. More high-quality randomised controlled trials are needed in the future to confirm the *in vivo* anti-tumour activity and survival benefit of metformin.

## Introduction

With advances in medical care, cancer mortality rates in high-income countries have improved significantly over the past few decades, reflecting the significant impact of income on the survival of cancer patients (1, 2). In terms of cancer incidence, the global incidence of cancer in men has shown a flat trend, while the incidence of cancer in women has increased slightly. However, in the 20 years between 1999 and 2019 cancer mortality rates increased by twice as much as in 1999, with more than 10 million people deaths from cancer in 2019 alone (3). Breast cancer has surpassed lung cancer as the highest detected cancer in women, followed by lung, colorectal, liver and stomach cancers (4). In contrast, prostate, lung and colon cancers are the most common in men. It is estimated that by 2050, there will be more than 6.9 million new cases of cancer in people over 80 years of age worldwide (5). The increasing incidence of cancer will undoubtedly increase the economic burden on patients, their families and society, and significantly affect the quality of life of patients and their families.

Metformin, as a first-line glucose-lowering drug for patients with type 2 diabetes (6), exerts its hypoglycaemic function mainly by reducing hepatic glucose output, increasing glucose uptake by whole-body muscle tissue and increasing tissue insulin sensitivity (7). Metformin can lower blood glucose and body weight to some extent. It is especially suitable for type 2 diabetic patients with obesity. Certainly, metformin does not only lower blood glucose and body weight, but can also improve e.g. NAFLD, metabolic diseases, inhibit inflammatory responses, exert cardiovascular protective effects, improve dyslipidaemia, regulate intestinal flora (8), and treat polycystic ovary syndrome (9). In addition, the use of metformin was found to be associated with a reduced risk of tumour development in patients with type 2 diabetes in retrospective studies (10–13). The results of meta-analyses has also shown that the use of metformin is associated with a reduced risk of cancer occurrence (14, 15). Diabetes is closely associated with the prognosis of cancer patients. Cancer patients with diabetes have higher rates of hospitalisation, length of stay and all-cause mortality compared to patients without diabetes (16). Diabetes was also associated with an increased risk of some neoplasms (17, 18). Some studies have shown that metformin use in cancer patients is associated with better prognostic outcomes, with metformin use extending progression-free survival and overall survival (19, 20). Although there are studies showing the anti-cancer effects of metformin. However, there are also studies doubting its anti-cancer effects, that is, the use of metformin is not associated with patient prognosis (21).

The anti-cancer effects of metformin were proposed in 2005 (22), which showed that long-term metformin use significantly reduced the risk of cancer in diabetic patients. As research developed, metformin also showed its antitumour activity *in vitro* and *in vivo (* 23). However, the mechanisms of anti-tumour action of metformin are still under intense research. The two key mechanisms that can be identified are direct inhibition of the AMPK/mTOR signalling pathway (24) and indirect anti-cancer activity through its hypoglycaemic and anti-inflammatory effects (25, 26).

The results above all suggest that metformin has an anti-cancer effect, but the ability of metformin to improve patient prognosis still needs to be confirmed in clinical trials. We also noticed that some of the studies included in the meta-analysis included patients with type 2 diabetes and did not reach conclusions in the clinical studies that were consistent with the retrospective studies. That is, the combination of metformin with adjuvant chemotherapy in patients without diabetes did not result in a significant benefit in terms of progression-free disease survival and overall survival. There are, of course, clinical studies that have reached conclusions consistent with the retrospective studies. Overall, it remains unclear whether the use of metformin can benefit cancer patients without diabetes. Therefore, the purpose of our study is to collect data from existing published and unpublished clinical studies to evaluate the efficacy and safety of metformin in adjuvant chemotherapy in cancer patients without diabetes and to provide additional data on whether the clinical use of metformin can improve patient prognosis.

## Methods

### Register

All methods for this systematic review and meta-analysis are outlined in a prospectively registered protocol available online(CRD42022353508), and reporting follows PRISMA (Preferred Reporting Items for Systematic Reviews and Meta-Analyses) guidelines.

### Eligibility criteria

Studies included in this meta-analysis were required to satisfy the following criteria: (i) the included studies were randomised controlled trials; (ii) the included studies were in cancer patients without diabetes but with no restrictions on cancer type or grade of cancer; (iii) the experimental group was treated with chemotherapy combined with metformin while the control group was treated with chemotherapy alone or chemotherapy combined with placebo; (iv) the primary outcomes were progression-free survival, overall survival and secondary outcome indicators included objective response rate, Clinical benefits, disease control rate and grade 3-4 adverse reactions. Progression-free survival and overall survival were reported using risk ratio (HR). Exclusion criteria: non-randomised controlled trials, inclusion of studies that did not rigorously exclude patients with diabetes, meeting abstracts, clinical trials that did not report outcomes, survival outcomes not described using a risk ratio (HR).

### Search strategy

Subject terms for metformin and Neoplasms as well as free terms were used to search relevant literature in Pubmed, Embase, The Cochrane Library, and references for selected reviews will also be searched. The search period is from the establishment of the library to August 2022. Further details of the search strategy are available in supplementary data(S1).

### Study selection

All retrieved studies were assessed for eligibility. Two reviewer first screened the results by title and abstract, and those who could not obtain valid information through the title and abstract for inclusion were read through in full text, and those studies for which full text was not available or results were not published were excluded. For studies that were published in multiple journals, we mainly selected the original published studies. Any queries were checked by a third reviewer and resolved by consensus.

### Data extraction

Data on patient characteristics, interventions, and outcomes for all studies were extracted into predesigned tables. These were cross-checked by a third independent reviewer and any disagreements were resolved by consensus. The extracted data mainly included patient’s age, number of patients, cancer type, treatment method, efficacy and adverse reaction related outcomes.

### Quality assessment

The Cochrane risk of bias assessment tool was used to determine the methodologic quality of RCTs.A total of 7 domains were evaluated: random sequence generation, allocation concealment, participant blinding, outcome assessor blinding, incomplete outcome data, selective reporting bias, and other sources of bias. The Cochrane risk of bias assessment tool permits each domain to be assessed and assigned a judgment of “low,” “high,” or “unclear” risk. Two reviewers independently performed these steps, and disagreements were resolved by a third reviewer or consensus-based discussion.

### Statistical analysis

Hazard ratio data could be obtained directly from the literature or by further analysis of the Kaplan-Meier curves. Revman5.4 software was used to analyze the pooled effect size. *I* ^2^ was used to evaluate the heterogeneity between studies. If *I* ^2^ < 50%, all studies were considered homogeneous and could be pooled. Otherwise, the effect size results were combined by random effect model. The *P* value was used to test the significance of the combined effect size. *P* value less than 0.05 was considered as a significant difference in the combined effect size; otherwise, there was no significant difference between the two groups. In addition, we will carry out subgroup analyses according to cancer type, tumour-based treatment modality, patient country and metformin dose, and thus study the efficacy of metformin use in cancer patients.

## Results

**Figure 1:**
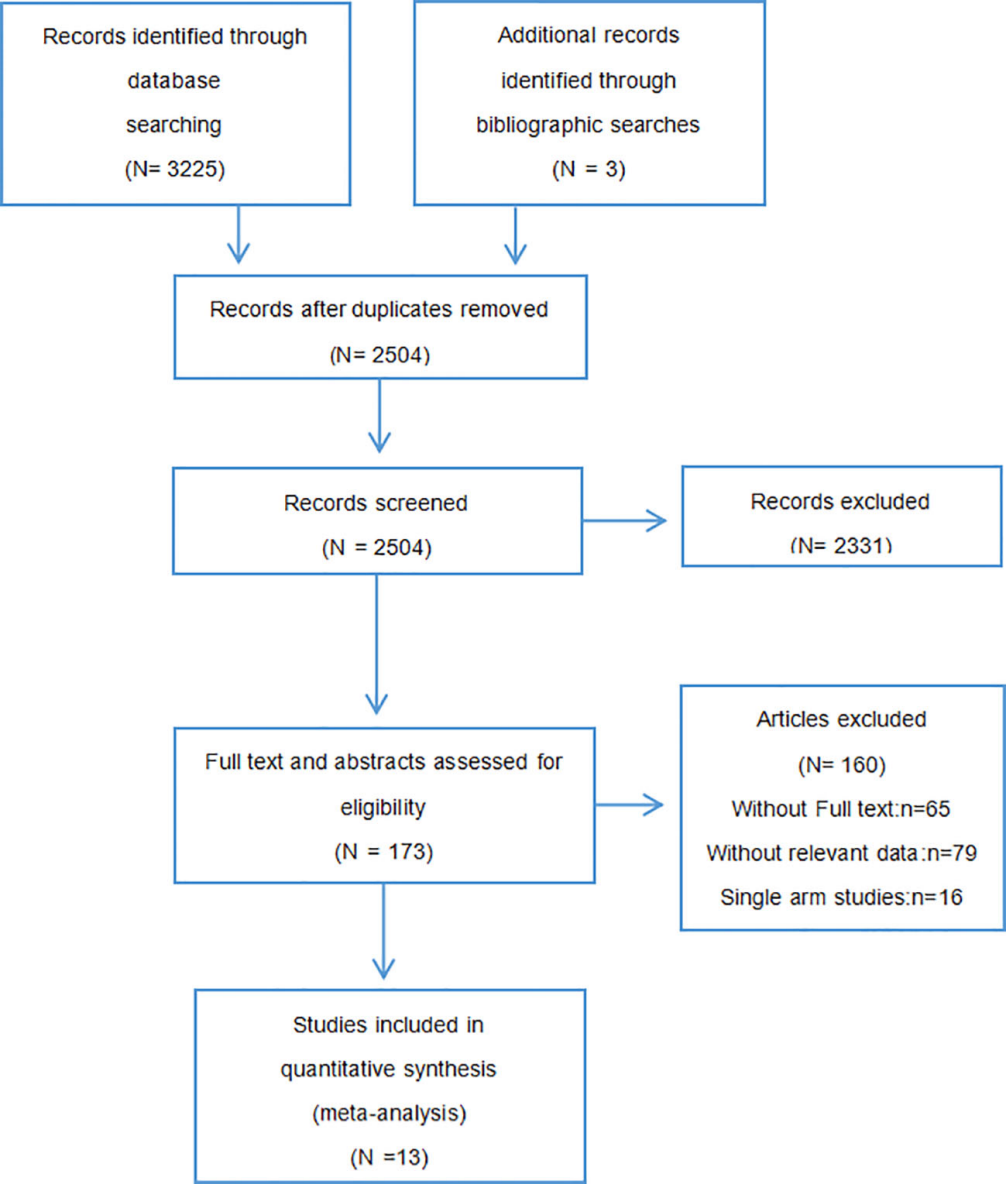
PRISMA study selection diagram.

**Figure 2:**
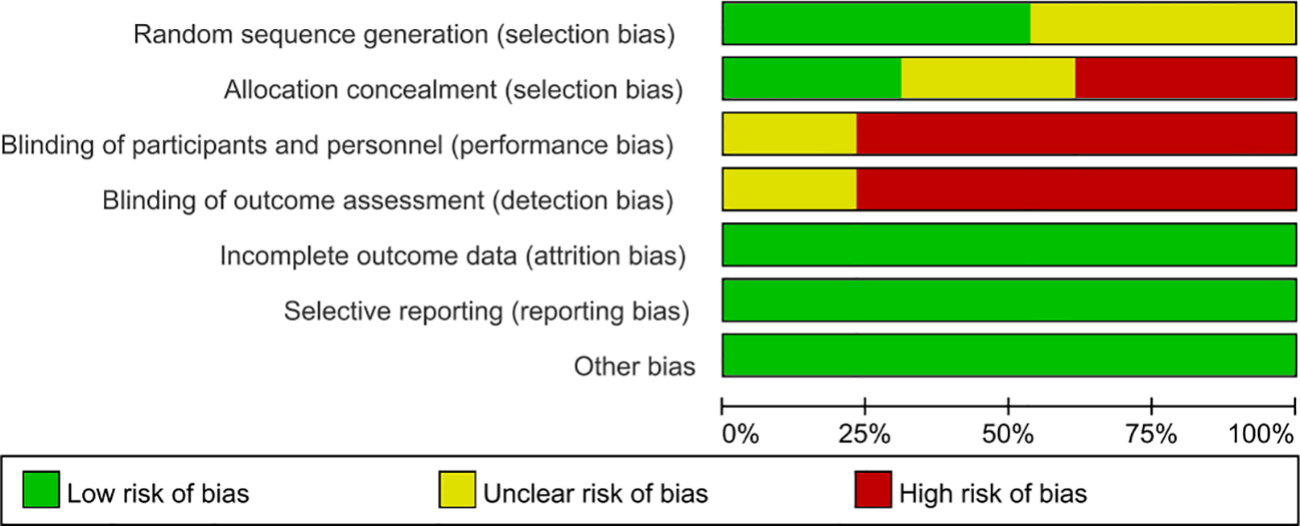
Risk of bias graph.

After screening 3228 records, the final 13 studies with a total of 1255 patients met our criteria, and all included studies were randomized controlled trials. Among them, 8 were non-small cell lung cancer (27–34), 4 were breast cancer (35–38), and 1 was prostate cancer (39). The literature screening process was shown in **Figure 1**. The general information of all the studies included in the meta-analysis is shown in **Table 1**. Publication bias is shown in **Figure 2** and **Supplementary Material S2**.

**Table 1:** The basic characteristics of the studies.

Author	Year	Tumor types	Sample size		Age		Outcome
			Experimental	Control	Experimental	Control	
Li Li	2022	Lung cancer	112	111	59.5(35-78)	59.0(32-76)	①②③④⑥
Oscar Arrieta	2019	Lung cancer	70	69	60.4(13.3)	58.4(1.6)	①②③④
T. Tsakiridis	2020	Lung cancer	26	24	65.9(8.1)	65.3(7.3)	①②
Skinner, H	2021	Lung cancer	86	81	/	/	①②⑥
KRISTEN A	2018	Lung cancer	19	6	58 (37-74)	64 (55-70)	①②
Rana Sayed	2015	Lung cancer	15	15	56 (44-70)	(37-76)	①③④
Guojunlan	2020	Lung cancer	60	60	54.6(6.2)	56.2(5.33)	②④
Fuyining	2021	Lung cancer	60	60	72.5(3.42)	72.0(3.0)	②④
Wangyaqi	2017	Breast cancer	30	30	67(46-78)	65(45-75)	O③④
O. Nanni	2018	Breast cancer	57	65	57(50-68)	61(54-66)	①②③⑥
Isabel Pimentel	2019	Breast cancer	22	18	55(39-75)	57(41-73)	①②④⑥
Yannan Zhao	2017	Breast cancer	30	30	57.5(33-72)	56.5(33-73)	①②③④⑥
Marc Pujalte	2021	Prostate cancer	50	49	70(54-84)	69(49-83)	①②③⑥

Note:①:Progression-Free-Surviva(PFS) ②:Overall Survival(OS) ③:Objective response rate(ORR) ④Disease control rate(DCR) ④ Clinical benefit rate(CBR) ⑥Grade 3-4 adverse reactions.

### Quality evaluation

The 13 randomised controlled trials were included in the Meta-analysis, 6 randomised controlled trials reported describing details of randomisation groups, the remaining 7 studies did not mention details of randomisation groups and 3 studies reported allocation concealment. In terms of blinding 10 studies were open-label studies and all studies had complete data; all studies specifically described interventions and outcome indicators; see **Figure 2**.

## Efficacy

### Progression-free survival

**Figure 3:**
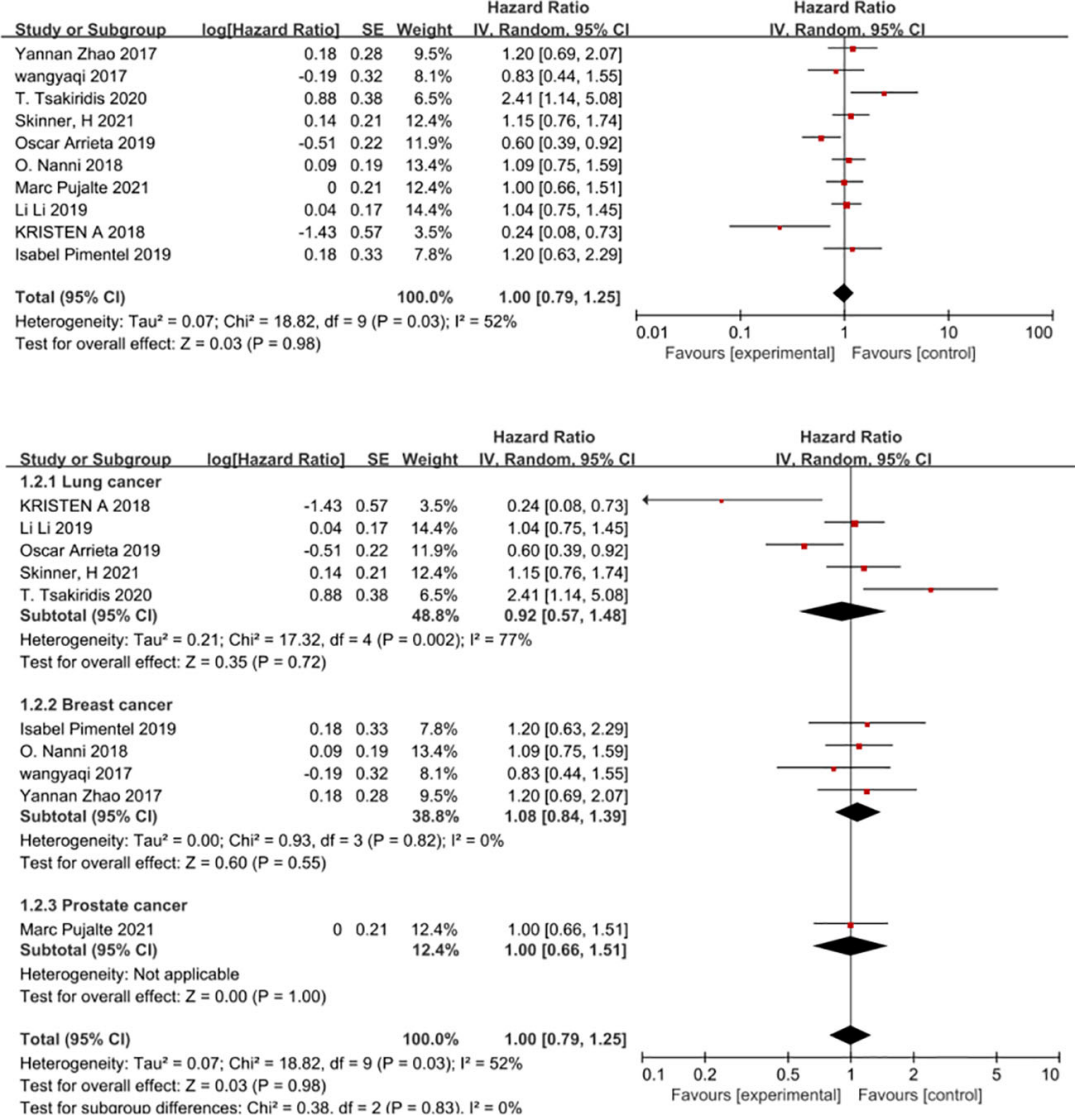
Progression-free survival.

A total of 10 studies reporting disease progression-free survival were included, of which 501 patients in the experimental group and 485 patients in the control group. Overall, the use of metformin did not benefit patients in terms of disease progression-free survival (HR=1,95CI 0.79-1.25), that is, the addition of metformin to cancer chemotherapy did not prolong patients’ progression-free survival (**Figure 3**). The results of the combined studies remained stable even with a fixed effects model for effect sizes, with the use of metformin not associated with prolonging patients’ tumour progression-free survival (HR=1.0, 95% CI 0.86-1.16, *I* ^2 ^= 52%). Begg’s test P=0.858, Egger’s test P=0.701 suggesting no significant publication bias in the studies. Because of the heterogeneity between studies, our subgroup analysis according to cancer type found that the addition of metformin to chemotherapy also did not prolong tumour progression-free survival in patients with a particular cancer (non-small cell lung cancer HR=0.92, 95% CI 0.57-1.48, breast cancer HR=1.08, 95% CI 0.84-1.39 and prostate cancer HR=1.0, 95%CI 0.66-1.51) (**Figure 3**). However, in the subgroup analysis according to cancer type metformin use was not found to be associated with improved tumour progression-free survival in patients with non-small cell lung cancer, but there was a high heterogeneity of results (*I* ^2 ^= 77%).

**Figure 4:**
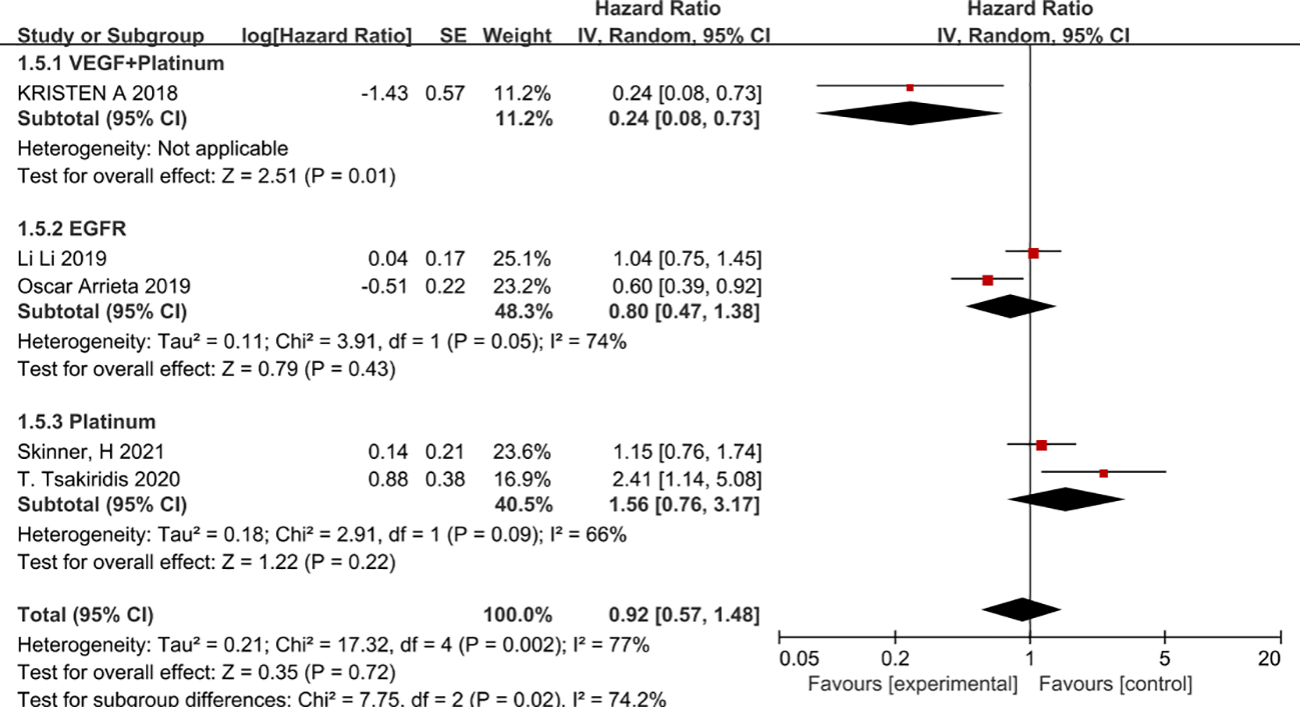
Chemotherapy regimens and progression-free survival.

**Figure 5:**
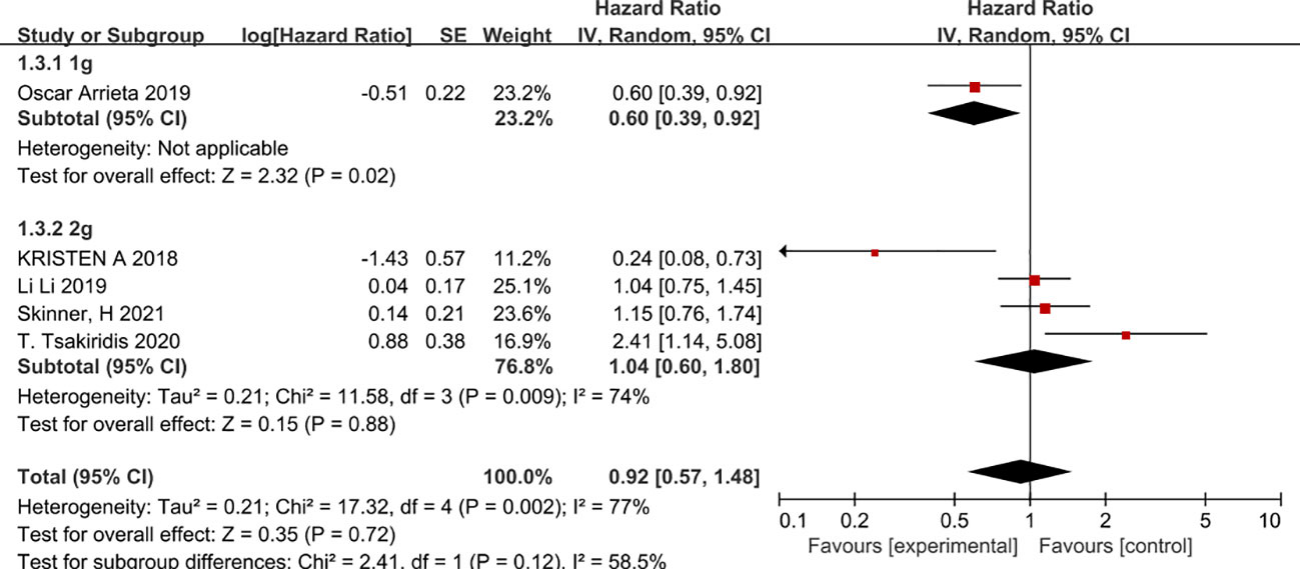
Metformin dose and progression-free survival.

**Figure 6:**
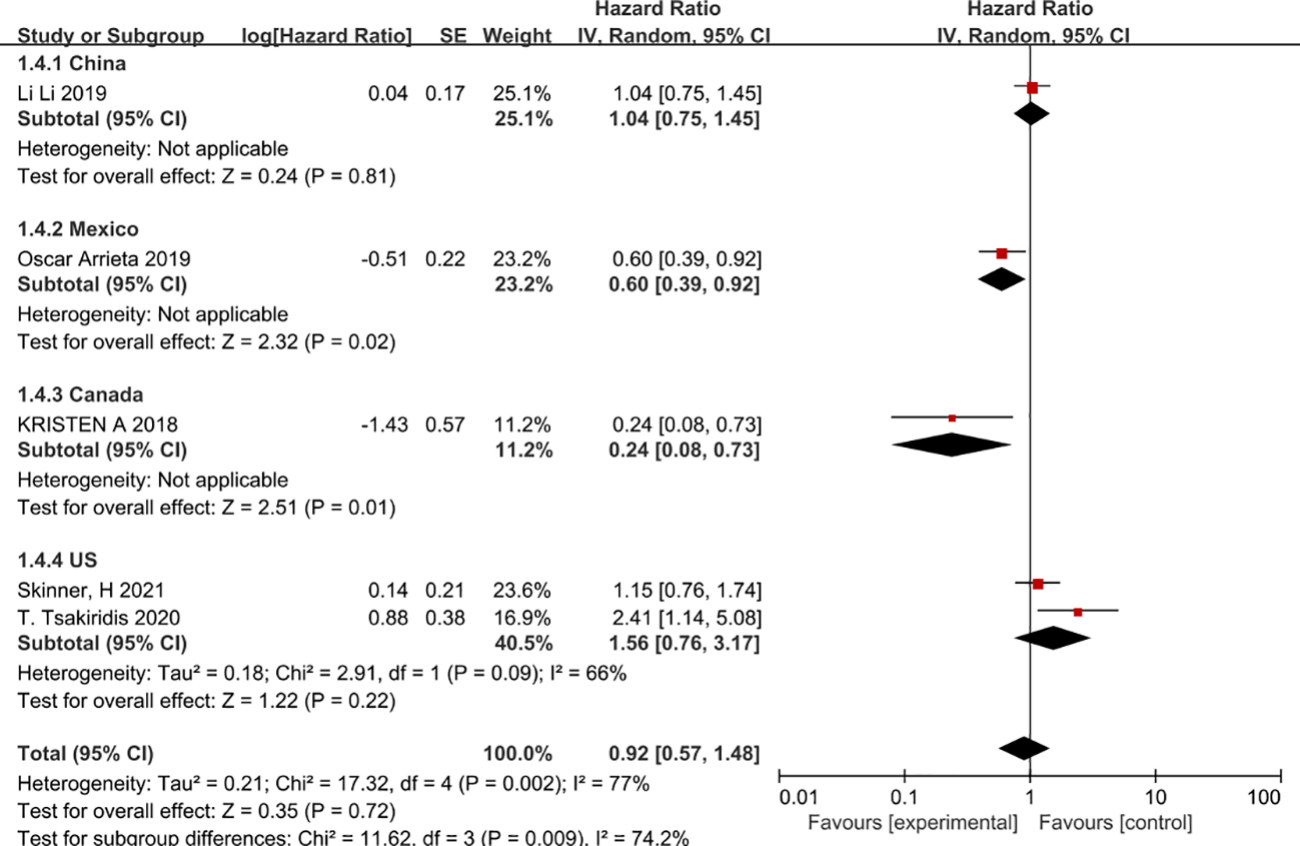
Patient country and progression-free survival.

In view of the high heterogeneity of metformin in improving progression-free survival in patients with non-small cell lung cancer, we conducted subgroup analyses in the following aspects. Firstly, in a subgroup analysis on chemotherapy regimens, combination chemotherapy regimens based on platinum-based or vascular endothelial growth factor (EGFR)-inhibitor-targeted agents did not benefit patients in terms of progression-free survival, whereas combination platinum-based regimens with vascular endothelial growth factor (EGFR) inhibitors improved patients’ progression-free survival (HR=0.24, 95% CI 0.08 -0.73) (**Figure 4**). The second was a subgroup analysis based on the dose of metformin that patients received in combination with chemotherapy; metformin at 2g per day did not benefit patients in chemotherapy, in that it prolonged patients’ disease-free survival. In contrast, 1 g of metformin per day combined with chemotherapy appeared to improve patients’ disease progression-free survival (HR=0.6, 95% CI 0.39-0.92) (**Figure 5**). The second was a subgroup analysis based on the dose of metformin that patients received in combination with chemotherapy; metformin at 2g per day did not benefit patients in chemotherapy, in that it prolonged patients’ disease-free survival. In contrast, 1 g of metformin per day combined with chemotherapy appeared to improve patients’ disease progression-free survival (HR=0.6, 95% CI 0.39-0.92 and HR=0.24, 95%CI 0.08-0.73), The benefit rate was higher in Canadian patients (**Figure 6**).

### Overall survival

**Figure 7:**
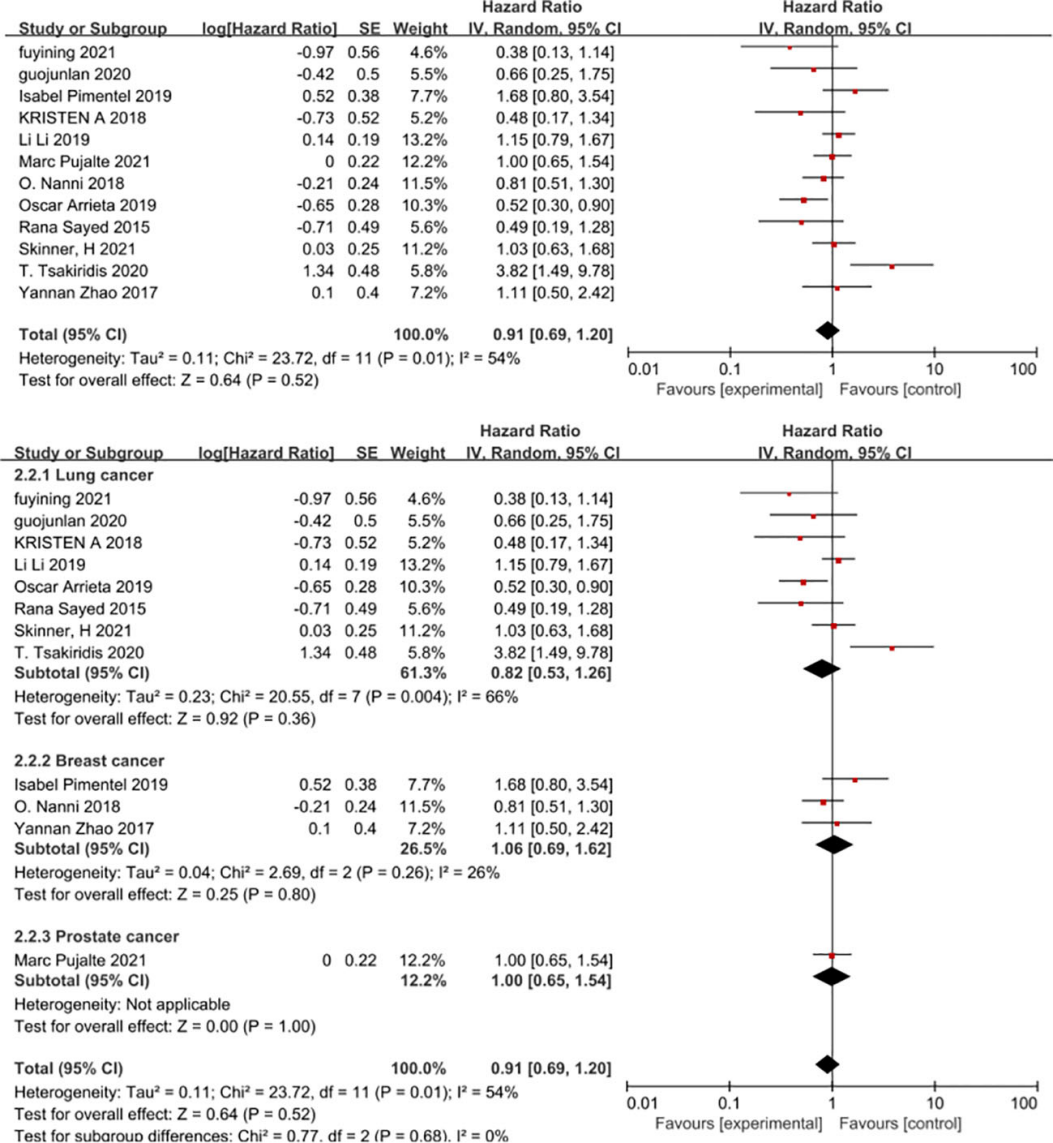
Overall survival.

Overall survival was reported in all 12 studies (27–37, 39) included in the meta-analysis. The results showed that the addition of metformin to chemotherapy in cancer patients without diabetes did not result in prolonged overall survival (HR=0.91, 95% CI 0.69-1.20). When the results were combined using a fixed effects model, the effect size combined results remained stable and metformin combined with chemotherapy did not prolong overall survival in cancer patients (HR=0.94, 95% CI 0.79-1.12, *I* ^2 ^= 54%) shown in **Figure 7**. Begg’s test P= 0.858 and Egger’s test P= 0.963, with no significant publication bias in the results. However, in a subgroup analysis according to cancer type we remained unable to find a benefit of metformin use for a specific cancer (non-small cell lung cancer HR=0.82, 95% CI 0.53-1.2, breast cancer HR=1.06, 95% CI 0.69-1.62, prostate cancer HR=1.0,95%CI0.65-1.54). Nevertheless, a subgroup analysis according to cancer type revealed a high heterogeneity in the amount of combined effects in patients with non-small cell lung cancer.

**Figure 8:**
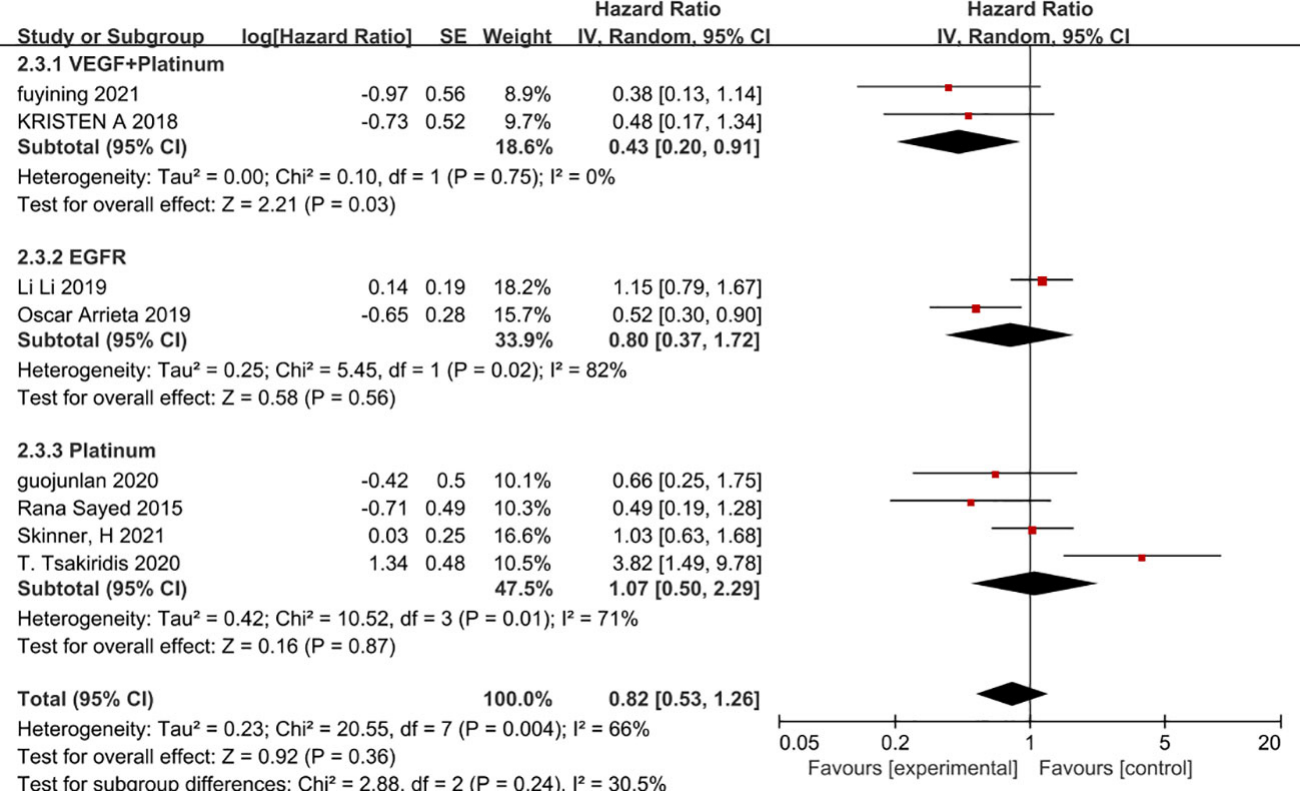
Chemotherapy Regimens and Overall Survival.

**Figure 9:**
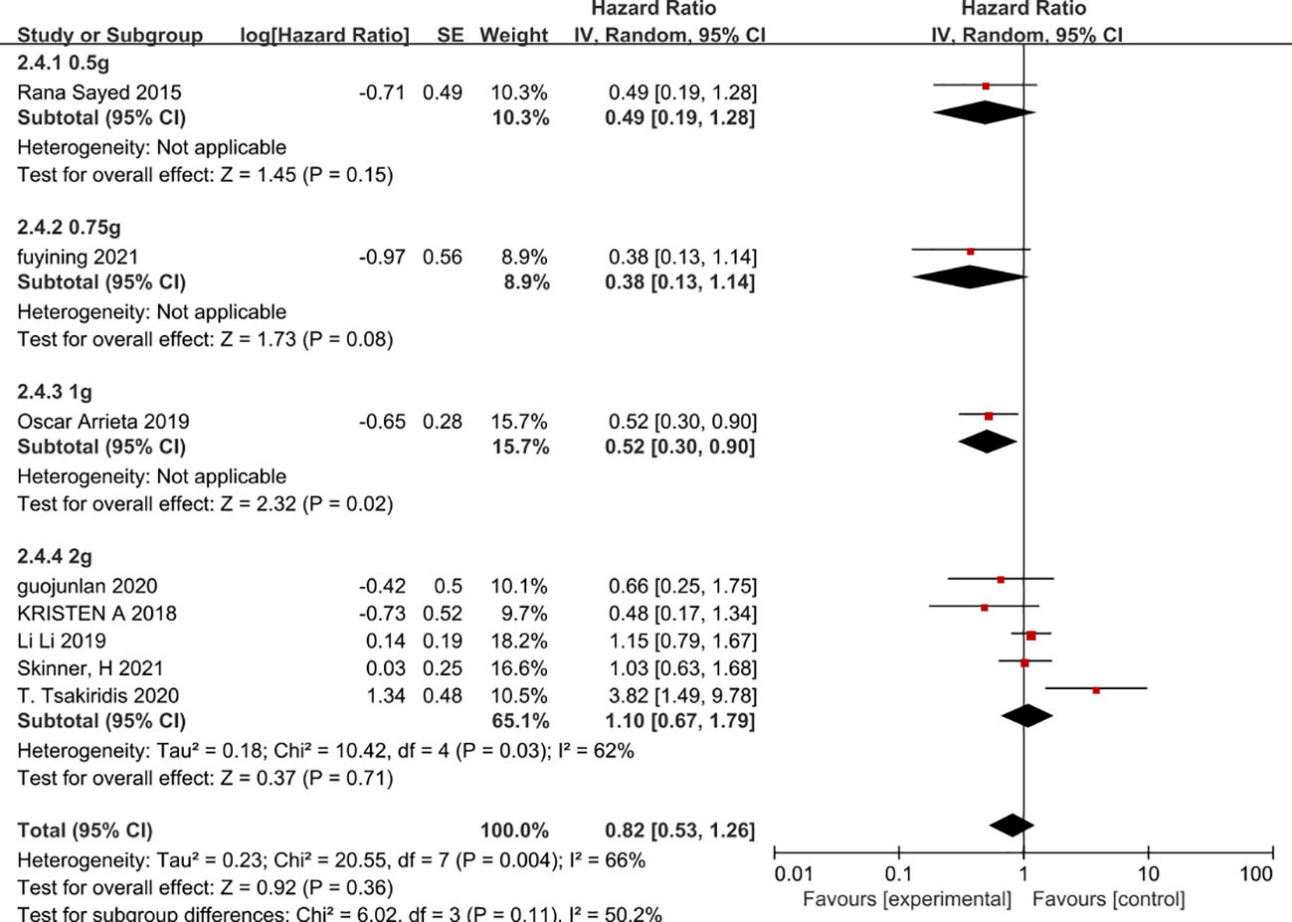
Metformin dose and overall survival.

**Figure 10:**
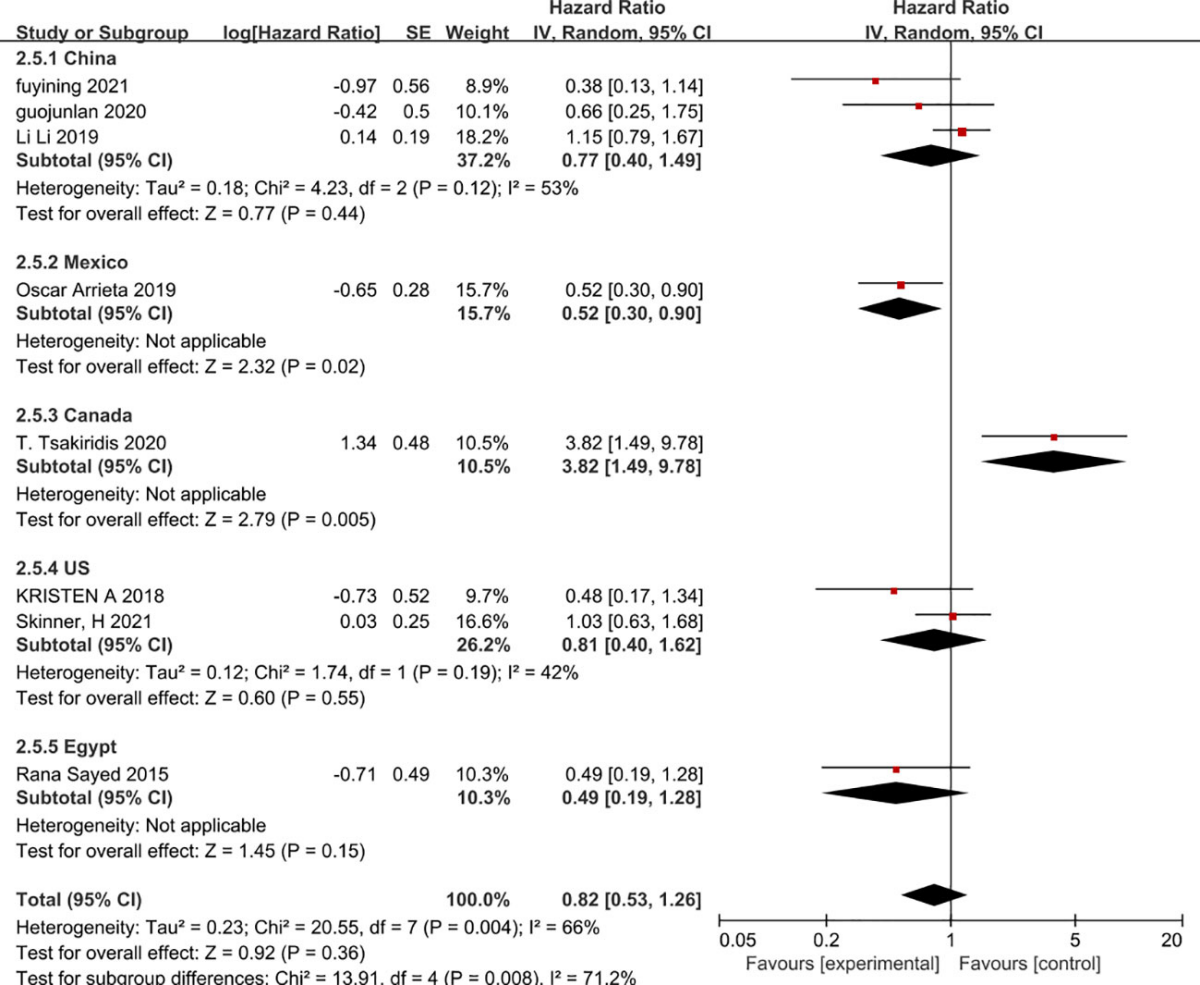
Patient country and overall survival.

Since there is a high level of heterogeneity between combined outcomes in patients with non-small cell lung cancer, we investigated this further by subgroup analysis. Subgroup analysis according to chemotherapy regimen showed that the effect of metformin on overall patient survival was independent of the chemotherapy regimen in which only VEGF inhibitors or platinum-based agents were used, whereas VEGF inhibitors combined with platinum-based agents benefited patients and prolonged overall patient survival (HR=0.43, 95% CI 0.2-0.91)(**Figure 8**). In addition, a subgroup analysis based on the dose of metformin in combination with chemotherapy showed that neither 0.5g, 0.75g nor 2g per day of metformin prolonged overall survival, whereas 1g per day of metformin was possible(HR=0.52, 95% CI 0.3-0.9) (**Figure 9**). Lastly, a subgroup analysis of the region in which the patients were studied showed that metformin did not benefit patients in terms of overall survival in China, the USA and Egypt, while in Canadian patients metformin use may even lead to premature death due to disease progression. In contrast, in Mexican patients, metformin use may have benefited overall survival and reduced the risk of death (HR=0.53, 95% CI 0.3-0.9) (**Figure 10**).

### Objective response rate

**Figure 11:**
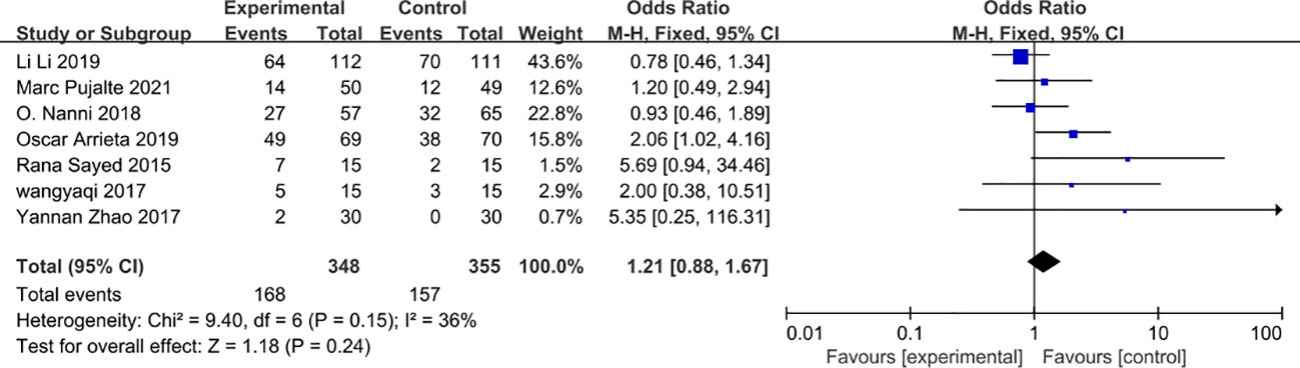
Objective response rate.

Objective response rates were evaluated in seven studies (27, 28, 31, 35, 37–39) with 348 patients in the experimental group 355 patients in the control group. Chemotherapy combined with metformin did not improve the objective response rate in cancer patients. Although there was some heterogeneity between studies (*I* ^2 ^= 36%) using a random effects model combined showed stable study results (OR=1.32, 95% CI 0.84-2.08, *I* ^2 ^= 36%) shown in **Figure 11**.

### Clinical benefits and disease control

**Figure 12:**
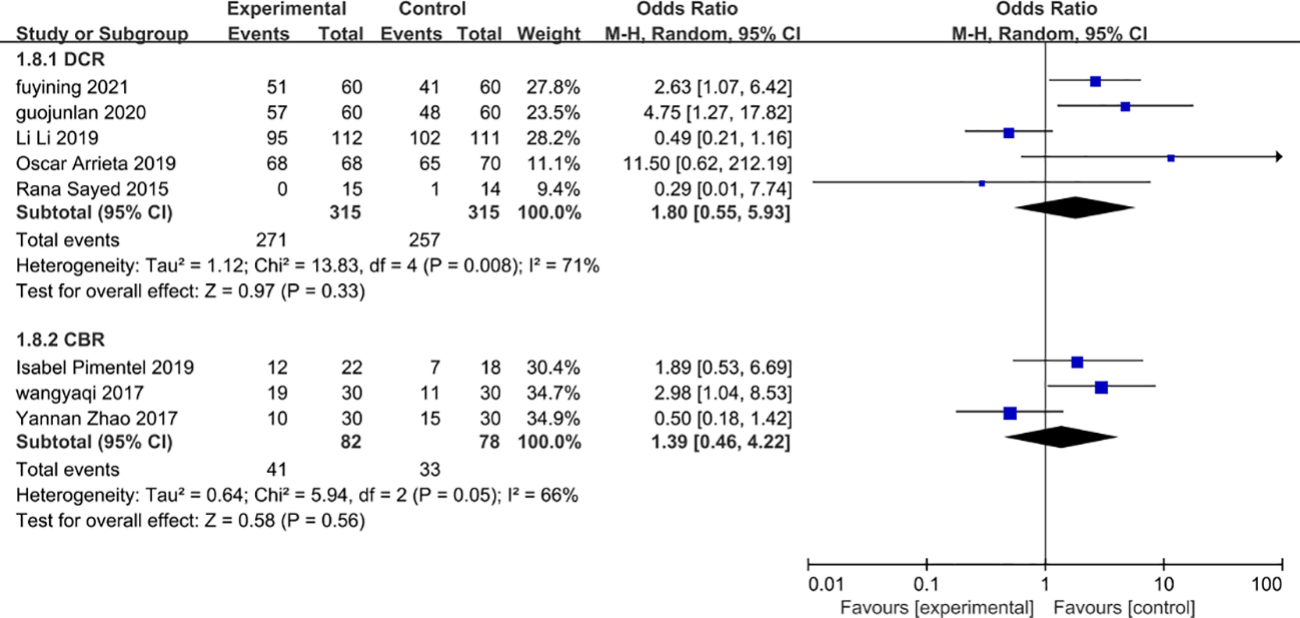
Clinical benefit rate and disease control rate safety.

Five studies (27, 28, 31, 33, 34) and three studies (35, 36, 38) were included in terms of disease control and clinical benefit, respectively. The use of metformin did not show an advantage in terms of clinical benefit or disease control rates (OR=1.80, 95% CI 0.55-5.93 and OR=0.92, 95% 0.13-6.34) and metformin in combination with chemotherapy did not increase clinical benefit rates or disease control rates in patients. However, there was a high degree of heterogeneity in both disease control and clinical benefit (**Figure 12**).

### Grade 3-4 adverse reactions

**Figure 13:**
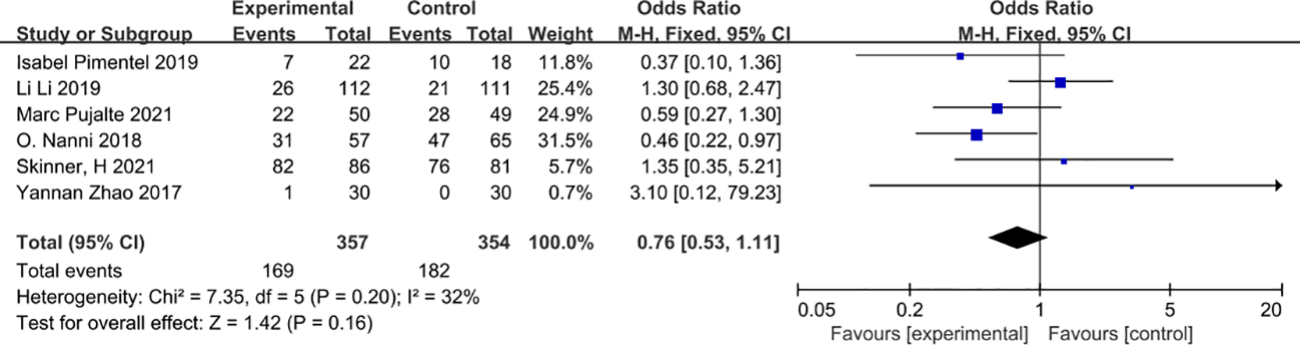
Grade 3-4 adverse reactions.

For safety six studies (27, 30, 35–37, 39) were included and there was no significant difference between the experimental group and the control group in terms of the occurrence of grade 3-4 serious adverse reactions (OR=0.76, 95% CI 0.53-1.1). Even after a combined effect size analysis using a random effects model the findings remained stable (OR=0.75, 95% CI 0.45-1.22, *I* ^2 ^= 32%) (**Figure 13**).

## Discussion

Our results show that the addition of metformin to adjuvant chemotherapy in cancer patients without diabetes does not prolong progression-free survival or overall survival. The role of metformin in cancer chemotherapy may be exaggerated, at least in our study which did not yield beneficial results for metformin in adjuvant chemotherapy in cancer patients without diabetes.

Although the results of some of the studies we included showed that metformin in combination with chemotherapy reduced the risk of death in patients, there were also some studies that showed that metformin use was associated with an increased risk of death in patients. The overall results of the studies showed that combining metformin during chemotherapy in patients without diabetes did not improve prognosis. In a subgroup analysis based on cancer typology, there was a high degree of heterogeneity in the results of use in patients with non-small cell lung cancer. In the subgroup analysis we found that chemotherapy regimens of vascular endothelial growth factor (VEGF) inhibitors in combination with platinum-based drugs in patients with non-small cell lung cancer and in Mexican and Canadian patients with 1 g of metformin daily prolonged progression-free survival to some extent. While 1 g of metformin daily prolonged overall survival in a subgroup analysis of overall survival, metformin use in Mexican patients may have benefited patients while in Canadian patients it may have led to an increased risk of death. Heterogeneity was highly stable across studies when analysing their heterogeneity, regardless of whether a fixed-effects or random-effects model was used.

In contrast, the systematic evaluation of the efficacy and safety of metformin in chemotherapy for cancer patients without diabetes in our study is a highlight of our study. However, in our study we produced results that were completely contradictory to the meta-analysis of the retrospective studies (15, 40), in that metformin did not benefit patients in chemotherapy for cancer patients without diabetes, either in patients with non-small cell lung cancer, breast cancer or prostate cancer, although there were no significant differences in terms of safety. Data on the efficacy of metformin were also reported in a phase 3 clinical trial that included 3649 breast cancer patients without diabetes, in which metformin did not show a significant difference in invasive disease-free survival compared with placebo (41). TAnother meta-analysis including seven randomised controlled trials also showed that metformin use did not prolong progression-free survival or overall survival in patients with advanced unresectable cancer, and there was no significant difference in grade 3-4 adverse effects, but this study included patients with diabetes (42). The same conclusion was reached in another phase 2 clinical study that included diabetic patients (43), however, the finding that metformin prolonged progression-free survival and overall survival in non-squamous cell carcinoma with high fluorodeoxyglucose uptake provides a potential direction for further research into metformin antitumour therapy. In addition, a secondary analysis of a study included in the meta-analysis (28) suggested that the use of metformin in patients with BMI >24 significantly prolonged progression-free survival as well as overall survival compared to patients with BMI ≤24, suggesting that BMI may be a factor influencing the efficacy of metformin in cancer chemotherapy (44).

There were also several limitations in our study as follows. Firstly, we did not include much literature, especially in breast and prostate cancers. Secondly, the number of patients in the studies we included was insufficient, which may have introduced some error in the results. Thirdly, the different regimens of chemotherapy included in the studies, with the existence of targeted agents in addition to metformin treatment as well as multiple conventional platinum-based chemotherapy regimens and radiotherapy combinations, may have allowed for differences in results between the studies. In addition, tumour staging varied between studies, which could also lead to bias in the results. Finally, there were differences in the dose of metformin used between study regimens, which may also have contributed to some differences in the study results.Therefore, in future studies, there is a need to design more high quality clinical trials with large samples to investigate the benefit potential of metformin in cancer chemotherapy based on tumour type, stage, species, chemotherapy regimen, metformin dose and patient BMI.

## Conclusion

In this meta-analysis of randomised controlled trial studies, we found that chemotherapy in combination with metformin in cancer patients without diabetes did not prolong progression-free survival and overall survival and improved disease control in patients, although there was no significant difference in terms of safety. More high-quality randomised controlled trials are needed in the future to confirm the *in vivo* anti-tumour activity and survival benefit of metformin.

## Data availability statement

The datasets presented in this study can be found in online repositories. The names of the repository/repositories and accession number(s) can be found in the article/**Supplementary Material**.

## Author contributions

KY proposed the research question and organized the relevant literature survey and the overall writing of the article. H-hL and KY conducted the literature search and data extraction and analysis. WZ provided methodological guidance and revised the article. All authors contributed to the article and approved the submitted version.

## References

[1] Arnold M Rutherford MJ Bardot A Ferlay J Andersson TM Myklebust T . Progress in cancer survival, mortality, and incidence in seven high-income countries 1995-2014 (ICBP SURVMARK-2): a population-based study. Lancet Oncol (2019) 20:1493–505. doi: 10.1016/S1470-2045(19)30456-5 PMC6838671 31521509

[2] Lortet-Tieulent J Georges D Bray F Vaccarella S . Profiling global cancer incidence and mortality by socioeconomic development. Int J Cancer (2020) 147:3029–36. doi: 10.1002/ijc.33114 32449164

[3] Lin L Li Z Yan L Liu Y Yang H Li H . Global, regional, and national cancer incidence and death for 29 cancer groups in 2019 and trends analysis of the global cancer burden, 1990-2019. J Hematol Oncol (2021) 14:197. doi: 10.1186/s13045-021-01213-z 34809683 PMC8607714

[4] Bray F Ferlay J Soerjomataram I Siegel RL Torre LA Jemal A . Global cancer statistics 2018: GLOBOCAN estimates of incidence and mortality worldwide for 36 cancers in 185 countries. CA Cancer J Clin (2018) 68:394–424. doi: 10.3322/caac.21492 30207593

[5] Pilleron S Soto-Perez-de-Celis E Vignat J Ferlay J Soerjomataram I Bray F . Estimated global cancer incidence in the oldest adults in 2018 and projections to 2050. Int J Cancer (2021) 148:601–8. doi: 10.1002/ijc.33232 PMC7754149 32706917

[6] Association AD . Pharmacologic approaches to glycemic treatment. Diabetes Care (2017) 40:S64–s74. 27979895 10.2337/dc17-S011

[7] Sciannimanico S Grimaldi F Vescini F De Pergola G Iacoviello M Licchelli B . Metformin: up to date. Endocr Metab Immune Disord Drug Targets (2020) 20:172–81. doi: 10.2174/1871530319666190507125847 31670618

[8] Zhou J Massey S Story D Li L . Metformin: an old drug with new applications. Int J Mol Sci (2018) 19. doi: 10.3390/ijms19102863 PMC6213209 30241400

[9] Street ME Cirillo F Catellani C Dauriz M Lazzeroni P Sartori C . Current treatment for polycystic ovary syndrome: focus on adolescence. Minerva Pediatr (2020) 72:288–311. doi: 10.23736/S0026-4946.20.05861-2 32418411

[10] Wang QL Santoni G Ness-Jensen E Lagergren J Xie SH . Association between metformin use and risk of esophageal squamous cell carcinoma in a population-based cohort study. Am J Gastroenterol (2020) 115:73–8. doi: 10.14309/ajg.0000000000000478 31821177

[11] Adalsteinsson JA Muzumdar S Waldman R Wu R Ratner D Feng H . Metformin is associated with decreased risk of basal cell carcinoma: a whole-population case-control study from Iceland. J Am Acad Dermatol (2021) 85:56–61. doi: 10.1016/j.jaad.2021.02.042 33610593

[12] Lee JW Choi EA Kim YS Kim Y You HS Han YE . Metformin usage and the risk of colorectal cancer: a national cohort study. Int J Colorectal Dis (2021) 36:303–10. doi: 10.1007/s00384-020-03765-x 32968891

[13] Kim HJ Lee S Chun KH Jeon JY Han SJ Kim DJ . Metformin reduces the risk of cancer in patients with type 2 diabetes: an analysis based on the Korean national diabetes program cohort. Med (Baltimore) (2018) 97:e0036. doi: 10.1097/MD.0000000000010036 PMC5841986 29465545

[14] Zhang Y Zhang Y Shi X Han J Lin B Peng W . Metformin and the risk of neurodegenerative diseases in patients with diabetes: a meta-analysis of population-based cohort studies. Diabetes Med (2022) 39:e14821. doi: 10.1111/dme.14821 35213749

[15] Ng CW Jiang AA Toh EMS Ng CH Ong ZH Peng S . Metformin and colorectal cancer: a systematic review, meta-analysis and meta-regression. Int J Colorectal Dis (2020) 35:1501–12. doi: 10.1007/s00384-020-03676-x 32592092

[16] Kiburg KV Ward GM Vogrin S Steele K Mulrooney E Loh M . Impact of type 2 diabetes on hospitalization and mortality in people with malignancy. Diabetes Med (2020) 37:362–8. doi: 10.1111/dme.14147 31559651

[17] Gong IY Cheung MC Read S Na Y Lega IC Lipscombe LL . Association between diabetes and haematological malignancies: a population-based study. Diabetologia (2021) 64:540–51. doi: 10.1007/s00125-020-05338-7 33409570

[18] Wojciechowska J Krajewski W Bolanowski M Kręcicki T Zatoński T . Diabetes and cancer: a review of current knowledge. Exp Clin Endocrinol Diabetes (2016) 124:263–75. doi: 10.1055/s-0042-100910 27219686

[19] Coyle C Cafferty FH Vale C Langley RE . Metformin as an adjuvant treatment for cancer: a systematic review and meta-analysis. Ann Oncol (2016) 27:2184–95. doi: 10.1093/annonc/mdw410 PMC5178140 27681864

[20] Al-Ghalib HA Al-Otaibi AD Tulaihi BA Al-Ghaleb S . The anti-proliferative role of metformin in non-diabetic female patients with breast cancer: systematic review and meta-analysis of randomized control trials. VM Media SP Zoo VM Group SK (2020). doi: 10.5603/DK.2020.0062

[21] Takiuchi T Machida H Hom MS Mostofizadeh S Frimer M Brunette LL . Association of metformin use and survival outcome in women with cervical cancer. Int J Gynecol Cancer (2017) 27:1455–63. doi: 10.1097/IGC.0000000000001036 PMC7526033 29049093

[22] Evans JM Donnelly LA Emslie-Smith AM Alessi DR Morris AD . Metformin and reduced risk of cancer in diabetic patients. Bmj (2005) 330:1304–5. doi: 10.1136/bmj.38415.708634.F7 PMC558205 15849206

[23] Dowling RJ Niraula S Stambolic V Goodwin PJ . Metformin in cancer: translational challenges. J Mol Endocrinol (2012) 48:R31–43. doi: 10.1530/JME-12-0007 22355097

[24] Dowling RJ Zakikhani M Fantus IG Pollak M Sonenberg N . Metformin inhibits mammalian target of rapamycin-dependent translation initiation in breast cancer cells. Cancer Res (2007) 67:10804–12. doi: 10.1158/0008-5472.CAN-07-2310 18006825

[25] Fidan E Onder Ersoz H Yilmaz M Yilmaz H Kocak M Karahan C . The effects of rosiglitazone and metformin on inflammation and endothelial dysfunction in patients with type 2 diabetes mellitus. Acta Diabetol (2011) 48:297–302. doi: 10.1007/s00592-011-0276-y 21424914

[26] Dowling RJ Goodwin PJ Stambolic V . Understanding the benefit of metformin use in cancer treatment. BMC Med (2011) 9:33. doi: 10.1186/1741-7015-9-33 21470407 PMC3224599

[27] Li L Jiang L Wang Y Zhao Y He Y . Combination of metformin and gefitinib as first-line therapy for nondiabetic advanced NSCLC patients with EGFR mutations: a randomized, double-blind phase II trial. Clin Cancer Res (2019) 25. clincanres.0437.2019. doi: 10.1158/1078-0432.CCR-19-0437 31413010

[28] Arrieta O Barrón F Padilla MS Avilés-Salas A Ramírez-Tirado LA Arguelles Jiménez MJ Vergara E Zatarain-Barrón ZL Hernández-Pedro N Cardona AF Cruz-Rico G Barrios-Bernal P Yamamoto Ramos M Rosell R. Effect of Metformin Plus Tyrosine Kinase Inhibitors Compared With Tyrosine Kinase Inhibitors Alone in Patients With Epidermal Growth Factor Receptor-Mutated Lung Adenocarcinoma: A Phase 2 Randomized Clinical Trial. JAMA Oncol. (2019) 5(11):e192553. doi: 10.1001/jamaoncol.2019.2553. 31486833 PMC6735425

[29] Tsakiridis T Pond G Wright J Ellis P Levine M . Randomized phase II trial of metformin in combination with chemoradiotherapy (CRT) in locally advanced non-small cell lung cancer (LA-NSCLC); the OCOG-ALMERA trial (NCT02115464). Int J Radiat OncologyBiologyPhysics (2020) 108:S104. doi: 10.1016/j.ijrobp.2020.07.2284

[30] Skinner H Hu C Tsakiridis T Santana-Davila R Lu B Erasmus JJ . Addition of metformin to concurrent chemoradiation in patients with locally advanced non-small cell lung cancer: the NRG-LU001 phase 2 randomized clinical trial. Am Med Assoc (2021). doi: 10.1001/jamaoncol.2021.2318 PMC8323052 34323922

[31] Sayed R Saad AS El Wakeel L Elkholy E Badary O . Metformin addition to chemotherapy in stage IV non-small cell lung cancer: an open label randomized controlled study. Asian Pacific J Cancer Prev (2015) 16:6621–6. doi: 10.7314/APJCP.2015.16.15.6621 26434885

[32] Marrone KA Zhou X Forde PM Purtell M Brahmer JR Hann CL . A randomized phase II study of metformin plus Paclitaxel/Carboplatin/Bevacizumab in patients with chemotherapy-naïve advanced or metastatic nonsquamous non-small cell lung cancer. Oncologist (2018) 23:859–65. doi: 10.1634/theoncologist.2017-0465 PMC6058336 29487223

[33] FuYining W . Effects of bevacizumab combined with concurrent chemoradiotherapy and metformin on the clinical effectiveness, immune function and tumor associated protein of NSCLC patients. J Xuzhou Med University (2021) 41:691–8.

[34] Guo J-L Zheng Yi Wang Y-X Wen Y-Y Wang H-H . The curative effect study of metformin combined with concurrent radiotherapy and chemotherapy in patients with non-small cell lung cancer. Chin J Rational Drug Use (2020) 17:63–6.

[35] Zhao Y Gong C Wang Z Jian Z Hu X . A randomized phase II study of aromatase inhibitors plus metformin in pre-treated postmenopausal patients with hormone receptor positive metastatic breast cancer. Oncotarget (2017) 8:84224–36. doi: 10.18632/oncotarget.20478 PMC5663590 29137418

[36] Ip A Ael A Me C Rjod B Dc B Cea B . A phase II randomized clinical trial of the effect of metformin versus placebo on progression-free survival in women with metastatic breast cancer receiving standard chemotherapy. Breast (2019) 48:17–23. doi: 10.1016/j.breast.2019.08.003 31472446

[37] Nanni O Amadori D De Censi A Rocca A Freschi A Bologna A . Metformin plus chemotherapy versus chemotherapy alone in the first-line treatment of HER2-negative metastatic breast cancer. the MYME randomized, phase 2 clinical trial. Breast Cancer Res Treat (2018). doi: 10.1007/s10549-018-05070-2 30536182

[38] Wang Y Wang M Dong H Qiu Z . Clinical observation of metformin in treatment of endocrine resistance and postmenopausal hormone receptor positive advanced breast cancer. J Pract Med (2017) 33:1377–80.

[39] Pujalte Martin M Borchiellini D Thamphya B Guillot A Paoli JB Besson D Hilgers W Priou F El Kouri C Hoch B Deville JL Schiappa R Cheli S Milano G Tanti JF Bost F Ferrero JM. TAXOMET: A French Prospective Multicentric Randomized Phase II Study of Docetaxel Plus Metformin Versus Docetaxel Plus Placebo in Metastatic Castration-Resistant Prostate Cancer. Clin Genitourin Cancer (2021) 19(6):501–9. doi: 10.1016/j.clgc.2021.08.008. 34629300

[40] Guo M Shang X Guo D . Metformin use and mortality in women with ovarian cancer: an updated meta-analysis. Int J Clin Pract (2022) 2022:9592969. doi: 10.1155/2022/9592969 35685604 PMC9159224

[41] Goodwin PJ Chen BE Gelmon KA Whelan TJ Ennis M Lemieux J . Effect of metformin vs placebo on invasive disease-free survival in patients with breast cancer: the MA.32 randomized clinical trial. Jama (2022) 327:1963–73. doi: 10.1001/jama.2022.6147 PMC9131745 35608580

[42] Gan X Cao C He Y Hu X Peng X Su Y . Metformin has no significant anticancer effect on patients with advanced or unresectable cancer: a systematic review and meta-analysis. Curr Pharm Des (2022) 28:1351–8. doi: 10.2174/1381612828666220329113434 35352646

[43] Lee Y Joo J Lee YJ Lee EK Park S Kim TS . Randomized phase II study of platinum-based chemotherapy plus controlled diet with or without metformin in patients with advanced non-small cell lung cancer. Lung Cancer (2021) 151:8–15. doi: 10.1016/j.lungcan.2020.11.011 33278671

[44] Arrieta O Turcott J Barrón F Ramos M Yendamuri S Zatarain Barron L . P48.09 body mass index predicts benefit from adding metformin to EGFR-TKIs in patients with lung adenocarcinoma: subanalysis from an RCT. J Thorac Oncol (2021) 16:S1109–S10. doi: 10.1016/j.jtho.2021.08.520

