# Supplementary material for: Efficacy and safety of metformin in combination with chemotherapy in cancer patients without diabetes: systematic review and meta-analysis
Source: Front Oncol. 2023 Jul 20;13:1176885. doi: 10.3389/fonc.2023.1176885 (PMC10402741; doi:10.3389/fonc.2023.1176885)
Supplement: Supplementary file 2 [file DataSheet_2.docx]

****Search details****

**Pubmed Search Strategy**

**((((((((((((((((((Tumor[Title/Abstract]) OR (Neoplasm[Title/Abstract])) OR (Tumors[Title/Abstract])) OR (Neoplasia[Title/Abstract])) OR (Neoplasias[Title/Abstract])) OR (Cancer[Title/Abstract])) OR (Cancers[Title/Abstract])) OR (Malignant Neoplasm[Title/Abstract])) OR (Malignancy[Title/Abstract])) OR (Malignancies[Title/Abstract])) OR (Malignant Neoplasms[Title/Abstract])) OR (Neoplasm, Malignant[Title/Abstract])) OR (Neoplasms, Malignant[Title/Abstract])) OR (Benign Neoplasms[Title/Abstract])) OR (Benign Neoplasm[Title/Abstract])) OR (Neoplasms, Benign[Title/Abstract])) OR (Neoplasm, Benign[Title/Abstract])) OR ("Neoplasms"[Mesh])) AND ((((((((Dimethylbiguanidine[Title/Abstract]) OR (Dimethylguanylguanidine[Title/Abstract])) OR (Glucophage[Title/Abstract])) OR (Metformin Hydrochloride[Title/Abstract])) OR (Hydrochloride, Metformin[Title/Abstract])) OR (Metformin HCl[Title/Abstract])) OR (HCl, Metformin[Title/Abstract])) OR ("Metformin"[Mesh]))**
